# Nitrite of Amyl in Chloral-Poisoning

**Published:** 1879-08

**Authors:** 


Nitrite of Amyl in Chloral-Poisoning.—Dr. J. G. Sinclair Coghill makes the following remarks upon the antidotal power of nitrite of amyl in chloral poisoning, in the *British Medical Journal*:

"The following case indicates so markedly the value of nitrite of amyl as an antidote in poisoning by chloral-hydrate, and otherwise presents so many other points of interest in connection with the symptoms produced by overdoses of that now much used and much abused drug, that I have yielded to the request of some of my medical brethren to make it public.

"A. B., aged 62, of spare habit, was a frequent and intense sufferer from gout, in seeking relief for which he unfortunately become somewhat addicted to alcoholic stimulants and narcotics generally. Late on the evening of the 23d of April, after a liberal potation of whisky, he took a large, but unfortunately an unascertained dose of his favorite anodyne, chloral. The dose must have been a very large one, for in a very few minutes he became completely insensible. Fortunately, medical assistance being at hand, the case received immediate attention from the very commencement of the symptoms, which, however, became so alarming that in about two hours I was sent for. I found that artificial respiration had been kept up for some time, but only with the effect of inducing feeble artificial, gasping respirations, at the rate of four per minute. The surface was cold and deeply cyanosed, and the pupils strongly contracted to the size of a pin's head. The pulse, however, was 80, full, but soft and compressible. I had the tongue at once pulled forward, and maintained in that position with forceps. Taking the state of the pupils as an indication, and remembering Liebreich's theory of the decomposition in the system of chloral into chloroform, I immediately administered by inhalation from a handkerchief about twenty drops of nitrite of amyl. The effect was immediate. Within two minutes warmth had returned, even to the extremities, and the surface had assumed the hue of health. Within ten minutes the respirations had become much deeper, reaching nine per minute, and afterwards gradually increased up to twelve. The amyl had to be repeated in a smaller dose in about two hours, with permanent effect. At 9.30 next morning, the general condition was found to have improved somewhat, but there was no return of consciousness; and an attempt to give fluid nourishment by mouth had produced great embarrassment of breathing. I ordered an enema of brandy and Liebig's extract in arrow-root to be given, and repeated every two hours. After the second enema the patient became quite sensible, recognized and spoke to those around him, and swallowed some food with little trouble. I saw him again at 6.30 P.M., when the water was drawn off in normal amount and quality. I am informed that he continued to improve until 9 P.M., when he suddenly started up as if from sleep, with staring eyes, threw up his hand, uttered a cry, and fell back dead. I am inclined to think this fatal result might possibly have been averted by a more copious and frequent stimulation *per anum.*

"The principal points of interest to be noted in this case are the extreme contraction of the pupils; the intense affection of the respiratory, the complete immunity of the circulatory system; the rapid recovery of warmth and color, with restoration of the respiratory function under the influence of nitrite of amyl; the return of consciousness in response to stimulation *per anum*, and the sudden failure of the heart's action, which proved immediately fatal.

"In cases of poisoning by chloral-hydrate, very opposite observations are on record with reference to the state of the pupils, and also as to the relative extent to which the action of the heart and lungs is influenced by the drug. Mr. W. Sedgwick, who has made a special study of the subject, states that in most instances the pupils are contracted; while Dr. Cleveland, and especially Dr. B. W. Richardson, report the contrary to be the invariable condition. I believe that the explanation of these apparently discrepant phenomena must be sought for in the difference in the amount of the drug swallowed, and the corresponding rapidity of its action. When chloroform is administered in excess *too rapidly*, it seems to prove fatal by paralyzing the respitory centres, while the pulse remains comparatively unaffected, the pupils being *contracted;* but when chloroform inhalation is kept up *too long*, so that the drug accumulates slowly in the system, the heart first yields to its influence, and succumbs earlier than the respiration, and under these circumstances the pupils will be found *dilated*. I have ascertained these conditions, both experimentally, in the lower animals, and from a large experience of chloroform administration, commenced 22 years ago, while assistant to the late Sir James Y. Simpson. A parallel discrepancy in symptoms may be noted in cases of *delirium tremens*, where the phenomena of the attack have been developed as a result of prolonged drinking to excess, or from one deep debauch.

"Liebreich, the discoverer of chloral-hydrate, believes that it acts on the system by being resolved into chloroform from decomposition in the presence of an alkali; and although this opinion is purely theoretical, yet it must be admitted that there are marked and close resemblances both in their physiological and therapeutic effects. This would at once explain why nitrite of amyl should be the best antidote in chloral poisoning, much more certainly than strychnia, which has been proposed as its antagonist; while, strangely enough, nitrite of amyl itself is proposed by Dr. B. W. Richardson as the antidote to strychnia poisoning. May it not be that nitrite of amyl will prove the appropriate antidote when the drug has been administered in such quantity as to act rapidly on the respiratory centres, *with contracted pupils*, and that strychnia should be given when the drug has acted slowly as a cumulative poison when the heart has succumbed, *and the pupils are found dilated?*

